# Molecular and Clinicopathological Differences by Age at the Diagnosis of Colorectal Cancer

**DOI:** 10.3390/ijms18071441

**Published:** 2017-07-05

**Authors:** Chu-Cheng Chang, Pei-Ching Lin, Chun-Chi Lin, Yuan-Tzu Lan, Hung-Hsin Lin, Chien-Hsing Lin, Shung-Haur Yang, Wen-Yi Liang, Wei-Shone Chen, Jeng-Kai Jiang, Jen-Kou Lin, Shih-Ching Chang

**Affiliations:** 1 Division of Colon & Rectal Surgery, Department of Surgery, Taipei Veterans General Hospital, Taipei 112, Taiwan; ccchang29@vghtpe.gov.tw (C.-C.C.); cclin15@vghtpe.gov.tw (C.-C.L.); ytlan@vghtpe.gov.tw (Y.-T.L.); hhlin7@vghtpe.gov.tw (H.-H.L.); yangsh@vghtpe.gov.tw (S.-H.Y.); wschen@vghtpe.gov.tw (W.-S.C.); jkjiang@vghtpe.gov.tw (J.-K.J.); jklin@vghtpe.gov.tw (J.-K.L.); 2 Department of Surgery, Faculty of Medicine, School of Medicine, National Yang-Ming University, Taipei 112, Taiwan; 3 Department of Clinical Pathology, Yang-Ming Branch, Taipei City Hospital, Taipei 11146, Taiwan; b7901127@tmu.edu.tw; 4 Division of Genomic Medicine, National Health Research Institutes, Zhunan 350, Taiwan; jameslin@fcbiotech.com.tw; 5 Department of Pathology, Taipei Veterans General Hospital, Taipei 112, Taiwan; wyliang@vghtpe.gov.tw; 6 Endoscopy Center for Diagnosis and Treatment, Taipei Veterans General Hospital, Taipei 112, Taiwan

**Keywords:** colorectal cancer, mutation, *p53*, *APC*, MSI, *PI3K*

## Abstract

We compared the clinicopathological and molecular profiles between different age groups of sporadic colorectal cancer (CRC) patients (age <50, 56–60, 60–70, 70–80, and >80); 1475 CRC patients were enrolled after excluding 30 individuals with Lynch syndrome. The mutation spectra for *APC*, *TP53*, *KRAS*, *PIK3CA*, *FBXW7*, *BRAF*, *NRAS*, *HRAS*, *TGFbR*, *Akt1*, and *PTEN* were analyzed using polymerase chain reaction (PCR), followed by MassArray and microsatellite (MSI-high) analysis by performing genotyping. Male patients (74.1%) were significantly predominant to females (25.9%) in the older age group (70–80, >80). There was an insignificantly linear trend between TNM staging and age-onset of CRC diagnosis. Patients aged < 50 had 58.7% diseases in the advanced stages (Stage III: 36.5% and IV: 22.2% respectively), while this decreased to 40.2% (Stage III: 26.2% and IV; 14.0% respectively) in patients >80. The distributions of mutation frequency were similar in majority of the genes studied among different age groups. Additionally, patients aged <50 had significantly higher frequency of MSI-high, *PTEN*, and *HRAS* mutations than those of other groups. Age-onset at diagnosis significantly affected overall survival (HR = 1.46; 95% CI: 1.35–1.58), but not cancer-specific survival (HR = 1.08; 95% CI: 0.99–1.18) in multivariate analysis. In conclusion, molecular and clinicopathological differences were not as significant among different age groups of CRC patients as previously suspected.

## 1. Introduction

Colorectal cancer (CRC) has been the most common cancer in Taiwan since 2006 [1]. As a disease predominantly affecting old individuals, 90% of all CRCs have been diagnosed in patients older than 50 years [2]. The postulated mechanisms of how aging impacts CRC carcinogenesis might include accumulation of somatic mutations and epigenetic silencing over time [3]. Data from a genome-wide association study also showed that the frequency of people with such clonal anomalies in a mosaic state is low, up to about 50 years of age, and then increases rapidly up to 2–3%. [4] Whether the molecular alterations driving colorectal cancer carcinogenesis differs by age at diagnosis, is not well known. [5].

However, the overall CRC incidence has decreased recently partly because of early CRC screening above the age of 50. Meanwhile, the worldwide prevalence of young-onset CRC is increasing owing to unidentified behavioral or environmental risk factors [6,7]. Moreover, most individuals with young-onset CRC are found to have a more advanced disease course and poor prognosis [7,8]. Young-onset CRCs have a high frequency of hereditary cancer syndromes such as Lynch syndrome, which is considered to be a less advanced disease with relatively good prognosis in CRC patients [9,10]. Since only 15% of young-onset CRCs is associated with Lynch syndrome, aggressive tumor phenotypes in other young-onset CRCs may be originating from sporadic mutations [11,12,13]. Recent study showed that sporadic young (age <45 years) CRC patients were common to have mutation in *FBXW7* but there were similar mutation rates of *TP53*, *KRAS* and *APC* between old and young-age cohorts [14].

Therefore, we hypothesized that molecular alterations would be disproportionately distributed between different age groups of CRC patients. In this study, we examined the mutation distributions of 12 genes and microsatellite instability according to age group (<50, 50–60, 60–70, 70–80, and >80 at diagnosis of CRC) among 1475 cases of CRC after exclusion of 30 CRC patients who had definite evidence of mismatch repair genes (MMR) germline mutation (Lynch syndrome).

## 2. Results

The patient population was composed of 970 men (65.8%) and 505 women (34.2%). The mean age at the time of tumor resection was 70.1 ± 11.5 years (range: 28–96 years; median: 72.2 years). There were 389 (26.4%) cases of proximal colon, 530 (35.9%) distal colon, and 556 (37.7%) rectal cancers. The histology showed poorly differentiated cancer, mucinous type, and lymphovascular invasion in 87 (5.9%), 130 (8.8%), and 287 (19.5%) patients, respectively. Only 17 (1.2%) cases had signet-ring cell carcinoma. The distribution of tumor staging (Tumor/Node/Metastasis, TNM) was stage I in 208 (14.1%), stage II in 552 (37.4%), stage III in 465 (31.5%), and stage IV in 250 (17.0%) patients. Of 1475 tumors, the most frequently mutated gene was *KRAS* (mutated in 589 cases, 39.9%), followed by *APC* (in 438, 29.7%), *TP53* (in 430, 29.2%), and *PIK3CA* (in 207, 14.0%). Additionally, 148 (10.0%) patients had cancers with (microsatellite) MSI-high.

**Figure 1:**
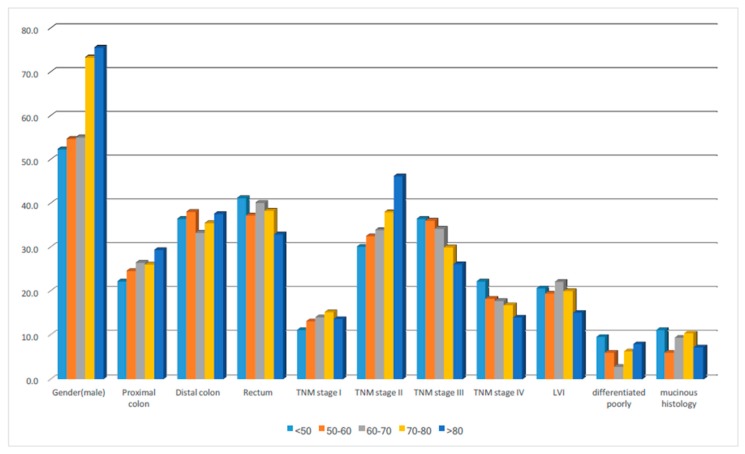
The clinicopathological distributions in different age groups of colorectal cancer patients.

As shown in Table 1 and Figure 1, the younger age groups of patients (<50, 50–60, 60–70) had a similar male-to-female ratio, but in older age groups of patients (70–80, >80) males were significantly predominant to females. There was an insignificantly linear association between disease staging and age onset of CRC (*p* = 0.083). Patients aged less than 50 had 58.7% diseases in advanced stages (Stage III: 36.5% and Stage IV: 22.2% respectively), but this decreased to 40.2% (Stage III: 26.2% and Stage IV: 14.0% respectively) in patients older than 80. The other pathological features and distribution of tumor locations were similar among these groups.

**Figure 2:**
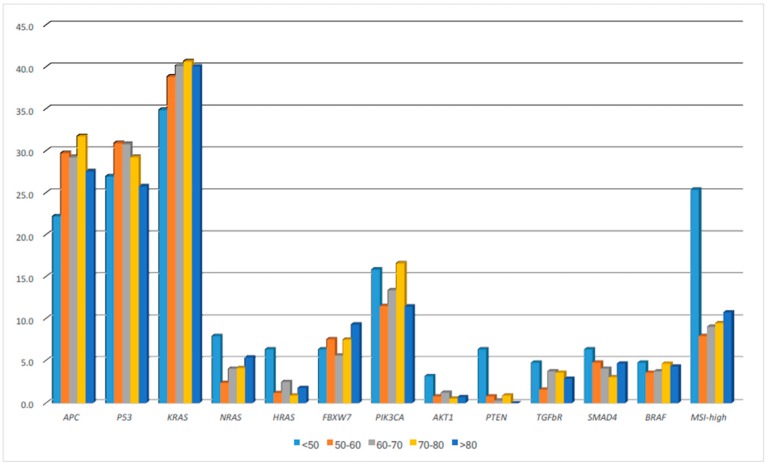
The distributions of molecular alterations in different age groups of colorectal cancer patients.

As shown in Figure 2 and Table 2, the distribution of mutation patterns between different age groups was almost similar except for higher *HRAS* and *PTEN* mutation. (*p* = 0.018, 0) in patients less than 50. The percentages of both *HRAS* and *PTEN* mutations was 6.3% in the age less than 50 group, whereas these two genes mutations were relatively rare and had similar distributions in CRC patients of other age groups.

**Figure 3:**
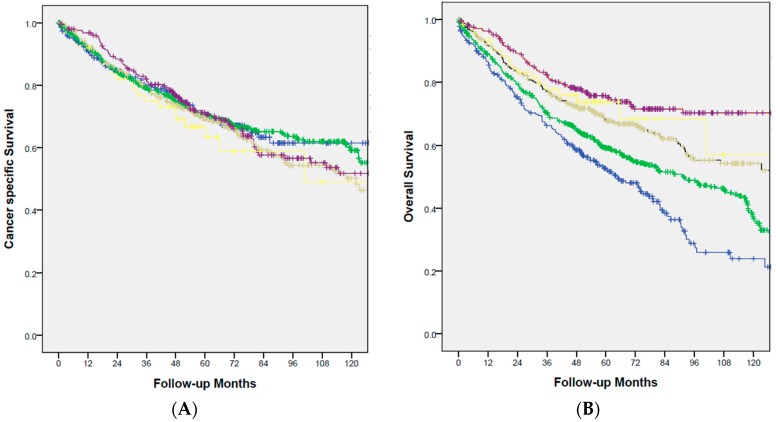
(**A**) Cancer-specific survival stratified by different age groups. (*p* = 0.731m, <50: yellow; 50–60: red; 60–70: gray; 70–89: green; >80: blue). (**B**) Overall survival stratified by different age groups. (*p* < 0.001; <50: yellow; 50–60: red; 60–70: gray; 70–89: green; >80: blue).

Within median follow-up of 61.3 months, 481 patients developed metastatic disease, including liver (271), lung (156), peritoneal (109), and others (54). The cancer-specific survival (CSS) and overall survival (OS) were showed in Figure 3. Although patients with younger CRC groups had more advanced disease, CSS were similar in these five groups of patients (*p* = 0.731, Figure 3A). The factors affecting CSS in multivariate analysis were TNM staging (HR = 4.56; 95% CI: 3.99–5.21), lymphovascular invasion (HR = 1.57; 95% CI: 1.27–1.88), and poor differentiation (HR = 1.60; 95% CI: 1.16–2.22) (Table 3). Overall survival was significantly affected by age at diagnosis of CRC (*p* < 0.001, Figure 3B). The multivariate analysis showed that TNM staging (HR = 2.04; 95% CI: 1.85–2.26), lymphovascular invasion (HR = 1.54; 95% CI: 1.28–1.86), and age at diagnosis (HR = 1.46; 95% CI: 1.35–1.58) were independent prognostic factors of overall outcome of CRC patients (Table 4). Although the outcome of patients with signet-ring cell carcinoma was very poor (median cancer-specific survival: 18.2 months), signet-ring cell carcinoma was not enrolled in multivariate analysis because of rare cases. (1.2%).

## 3. Discussion

Our results made three major contributions: first, sporadic old age-onset CRC occurred predominantly in males. Second, there was a linear trend showing that younger patients had a higher proportion of advanced disease, but with statistical insignificance. Third, molecular features were similar between different age groups of CRC patients, besides higher MSI-high and higher *PTEN* and *HRAS* mutations in CRC patients aged less than 50.

Increasing incidences of CRC in patients under 50 years of age have been observed recently through large-scale epidemiological analysis, including surveillance, epidemiology, and end results as well as smaller institutional reviews [3,15,16]. Possible reasons for the increasing trend in young-onset CRC have been proposed; however, these reasons are inconclusive because of great differences in lifestyles, including intake of a diet high in red or processed meat and low in fiber or vegetables and fruit, less physical activity, alcohol consumption, and smoking [17,18,19,20,21,22]. These changes in lifestyle have been linked to obesity and cancer development. For example, dysregulation of insulin-IGF-1 axis, sex hormones, or adipokines in obese individuals might result in multiple systemic metabolic alterations, such as insulin resistance, hyperglycemia, and chronic inflammation that can contribute to increased cancer risk in such patients [23,24]. Recent study provided the evidence that *KRAS*-mutated CRC but not *KRAS*-wild CRC had lower level of adiponectin, one of adipokines, which played important role in energy homeostasis [25].

The molecular signatures of young-onset CRCs are well defined in hereditary CRC cases, and a germline mutation in a cancer susceptibility gene is identified [9,10]. After excluding 30 Lynch syndrome cases, sporadic patients with CRC in our series shared similar mutation spectra in most CRC-associated genes between different age groups of patients, besides *PTEN* and *HRAS* mutation. Previous studies showed that *PTEN* mutation carriers show an increased incidence of colorectal adenocarcinomas (up to 13%), all occurring under the age of 50 [26]. Our study showed that *PTEN* mutation was found only in the tumors of young patients (4/63), but they did not have any family history of colon or other cancer history. Overall, our series showed that only 1.7% of CRC had *HRAS* mutations, compatible with findings of previous studies [27]. Of these, 25 *HRAS* mutations were all identical as *Q61L*, a hot spot mutation site in variable types of cancers. Although the *HRAS Q61L* mutation resulted in allosteric changes in the structure of *HRAS* in regions distal from the site of the mutation [28], the prognostic value in cancer and downstream effects of *HRAS Q61L* mutation were inconclusive. [29] Interestingly, patients aged less than 50 had higher frequency of *HRAS* mutation (6.3%) than those of other age groups (0.9–2.5%). Since frequency of *PTEN* and *HRAS* mutation was low in CRC, the role of these two genes in colorectal carcinogenesis in young patients needed to be studied.

Our series showed that there was no significant difference in a majority of the genes between different age groups. As previous studies, the mutation rate of *APC*, *TP53* and *KRAS* were similar in different age group [5,14]. However we did find that mutation frequency of *APC* and *KRAS* was a little lower in patients aged less than 50 than those of other groups, but there was a significantly higher frequency of MSI-high in young-age patients. The possible causes might be there was an undetected Lynch syndrome in young-age patients, or CpG island methylator phenotype (CIMP) was not so rare in young age patients. In contrast to some studies suggesting that promoter methylation of CpG islands (CIMP-H) plays a minor role in the neoplastic progression of early onset of colorectal carcinoma due to rare *BRAF* mutations in young patients [11,30], our study showed that young patients still had 4.8% incidence of *BRAF* mutation, which was similar to those of other age groups. This result indicated that the CIMP pathway could not be overlooked in young patients. Since mutation patterns were similar between different age CRC patients, a common earlier event in CRC should exist. For example, genome-wide DNA hypomethylation could contribute to genomic instability and act as an initiator of CRC [31,32,33,34]. Also LINE-1 hypomethylation, a surrogate marker of genome-wide hypomethylation, is a unique feature of early-onset CRC patients [35]. In MSI-high CRC patients, LINE-1 hypomethylation was found to be associated with more dismal outcome than MSS CRC patients [36].

Similar to other studies, we found that there was a trend that young-onset patients had higher proportions of advanced disease. The possible causes might be that young-onset CRC patients take longer time for diagnosis and longer symptom duration and workup duration than old patients [37,38,39]. However, young patients showed longer overall survival because they had a relatively good healthy condition and a keen desire to receive advanced chemotherapy, which was reflected by similar CSS between different age patients. This assumption had its limitation because our database did not have details of chemotherapy regimens in each individual, we could not know whether younger CRC patients received more aggressive chemotherapy.

Besides the family history available in our database, the lack of data on dietary habits, alcohol intake, and smoking history, physical activity, and body mass index of the patients was a limitation of this study. However, the molecular panel included most common mutation sites and microsatellite instability. Our results could not support our hypothesis that molecular alterations were disproportionately distributed between different age groups of CRC patients, but they indicated that sporadic CRC had similar molecular alterations between different age groups of CRC patients except for *PTEN* and *HRAS* mutation. Therefore, the upstream initiator involved in CRC development needs to be clarified in early onset CRC.

## 4. Materials and Methods

### 4.1. Clinical Data

In total, 1475 CRC patients underwent surgery at the Taipei Veterans General Hospital from 2000 to 2010 (Approval by the Institutional Review Board of Taipei Veterans General Hospital (number 2013-04-042B) [40,41,42]. Exclusion criteria were preoperative radio chemotherapy, emergency operations, or death within 30 days of surgery. Thirty patients that had definite germline mutation of MMR genes were also excluded. Clinical information that had been prospectively obtained and stored in the database included age, sex, personal and family medical history, location of tumor, TNM stage, differentiation, pathological prognostic features, and follow-up conditions. The colon length between the cecum and splenic flexure colon was defined as proximal colon. The distal colon was extended from the splenic flexure to the rectosigmoid colon. The rectum was within 15 cm of the anal verge.

Following surgery, patients were monitored every three months for the first two years and semiannually thereafter. The follow-up protocol included physical examination, digital rectal examination, carcinoembryonic antigen analysis, chest radiography, abdominal sonogram, and computerized tomography, if needed. Proton emission tomography or magnetic resonance imaging was arranged for patients with elevated levels of carcinoembryonic antigen but an uncertain site of tumor recurrence.

### 4.2. Collection of Tumor Tissues

Before sample collection, written informed consent for tissue collection was obtained from all patients. Samples were meticulously dissected and collected from different quadrants of the tumors, snap frozen in liquid nitrogen, and stored at Taipei Veterans General Hospital Biobank.

### 4.3. DNA Isolation and Quantification

After approval by the Institutional Review Board of Taipei Veterans General Hospital (number 2013-04-042B), samples for this study were obtained from the Biobank (Taipei Veterans General Hospital). DNA was extracted using the QIAamp DNA Tissue Kit (Qiagen, Valencia, CA, USA) according to the manufacturer’s recommendations. The quality of DNA was confirmed using Nanodrop 1000 Spectrophotometer (Thermo Scientific, Waltham, MA, USA)).

### 4.4. MassArray-based Mutation Characterization

The MassDetect CRC panel (v2.0), enabling the identification of 139 mutations in 12 genes, was selected from hotspots found in previous studies and the COSMIC database [43,44]. The polymerase chain reaction (PCR) and extension primers for the mutations were designed using MassArray Assay Design 3.1 software (Sequenom, San Diego, CA, USA). The details of the MassArray-based mutation detection methods are described in Supplementary file. The PCR products of the multiplexed reactions were spotted onto SpectroCHIP II arrays, and the DNA fragments were resolved on MassArray Analyzer 4 System (Sequenom). Each spectrum was then analyzed using Typer 4.0 software (Sequenom) to identify mutations. In this study, we defined 5% abnormal signal as a putative mutation. Putative mutations were then filtered by manual review. Any detected mutation in *BRAF*, *KRAS*, and *NRAS* was selected to be confirmed by Sanger sequencing. The concordance of MassArray and Sanger sequencing was 99.1%.

### 4.5. MSI Analysis

According to international criteria, five reference microsatellite markers were used for the determination of MSI: *D5S345*, *D2S123*, *BAT25*, *BAT26*, and *D17S250*. The primer sequences for these genes were obtained from GenBank (www.gdb.org). The detection of MSI was performed as previously described [45]. Samples with ≥2 MSI markers were defined as having high MSI, and those with 0–1 MSI markers were classified as microsatellite stable.

### 4.6. Statistical Analysis

The statistical endpoint for cancer specific survival (CSS) and overall survival (OS) was measured from the date of surgery. Patients not known to have died were censored at the date of last follow-up. Kaplan-Meier survival curves were plotted and compared using the log-rank test. The impact of clinicopathological features on CSS and OS was assessed using Cox regression univariate and multivariate analyses. The chi-square test and 2-tailed Fisher’s exact procedure were used to compare the genotype frequency of clinicopathological features. The numerical values were compared using the student’s *t*-test. Data were expressed as mean ± standard deviation. Statistical significance was defined as *p* < 0.05. Statistical analyses were performed using SPSS for Windows (version 16.0).

## 5. Conclusions

Molecular and clinicopathological differences were not as significant among different age groups of CRC patients as previously suspected. Sporadic CRC had similar molecular alterations between different age groups of CRC patients except for *PTEN* and *HRAS* mutation.

## Figures and Tables

**Table 1:** Clinicopathological difference between different age groups of CRC (colorectal cancer) patients.

Variable	<50	%	50–60	%	60–70	%	70–80	%	>80	%	*p*
Case No./%	63	4.3	252	17.1	321	21.8	560	38.0	279	18.9	
Gender(male)	33	52.4	138	54.8	177	55.1	411	73.4	211	75.6	<0.001
Location											
Right	14	22.2	62	24.6	85	26.5	146	26.1	82	29.4	0.719
Left	23	36.5	96	38.1	107	33.3	199	35.5	105	37.6	
Rectum	26	41.3	94	37.3	129	40.2	215	38.4	92	33.0	
TNM stage											
I	7	11.1	33	13.1	45	14.0	85	15.2	38	13.6	0.083
II	19	30.2	82	32.5	109	34.0	213	38.0	129	46.2	
III	23	36.5	91	36.1	110	34.3	168	30.0	73	26.2	
IV	14	22.2	46	18.3	57	17.8	94	16.8	39	14.0	
LVI	13	20.6	49	19.4	71	22.1	112	20.0	42	15.1	0.281
Poor differentiation	6	9.5	15	6.0	9	2.8	35	6.3	22	7.9	0.058
Mucinous histology	7	11.1	15	6.0	30	9.3	58	10.4	20	7.2	0.223

LVI: lymphovascular invasion.

**Table 2:** Number and frequency of molecular alterations in different age groups of CRC patients.

Gene	<50	%	50–60	%	60–70	%	70–80	%	>80	%	*p*
Case No/%	63	4.3	252	17.1	321	21.8	560	38.0	279	18.9	
*APC*	14	22.2	75	29.8	94	29.3	178	31.8	77	27.6	0.482
*P53*	17	27.0	78	31.0	99	30.8	164	29.3	72	25.8	0.645
*KRAS*	22	34.9	98	38.9	129	40.2	228	40.7	112	40.1	0.92
*NRAS*	5	7.9	6	2.4	13	4.0	23	4.1	15	5.4	0.263
*HRAS*	4	6.3	3	1.2	8	2.5	5	0.9	5	1.8	0.018
*FBXW7*	4	6.3	19	7.5	18	5.6	42	7.5	26	9.3	0.537
*PIK3CA*	10	15.9	29	11.5	43	13.4	93	16.6	32	11.5	0.184
*AKT1*	2	3.2	2	0.8	4	1.2	3	0.5	2	0.7	0.272
*PTEN*	4	6.3	2	0.8	1	0.3	5	0.9	0	0.0	<0.001
*TGFbR*	3	4.8	4	1.6	12	3.7	20	3.6	8	2.9	0.513
*SMAD4*	4	6.3	12	4.8	13	4.0	17	3.0	13	4.7	0.564
*BRAF*	3	4.8	9	3.6	12	3.7	26	4.6	12	4.3	0.945
MSI-high	16	25.4	20	7.9	29	9.0	53	9.5	30	10.8	<0.001

**Table 3:** Multivariate analysis for cancer specific survival.

Variable	Hazard Ratio	95% Confidence Interval	*p*
TNM	4.56	3.99–5.21	<0.001
Lymphovascular invasion	1.57	1.27–1.88	<0.001
Poorly differentiated	1.60	1.16–2.22	0.004
Mucinous histology	1.15	0.86–1.54	0.339
Age classification	1.08	0.99–1.18	0.075

**Table 4:** Multivariate analysis for overall survival.

Variable	Hazard Ratio	95% Confidence Interval	*p*
TNM	2.04	1.85–2.26	<0.001
Lymphovascular invasion	1.54	1.28–1.86	<0.001
Poorly differentiated	1.29	0.95–1.75	0.106
Mucinous histology	1.19	0.93–1.53	0.166
Age classification	1.46	1.35–1.58	<0.001

## Abbreviations

APC	Adenomatous Polyposis Coli
BRAF	B-Raf proto-oncogene
CSS	Cancer Specific Survival
CRC	Colorectal cancer
CI	Confidence interval
CIMP	CpG island methylator phenotype
FBXW7	F-box and WD repeat domain containing 7
KRAS	KRAS proto-oncogene
LVI	Lymphovascular invasion
MSI	Microsatellite instability
OS	Overall survival
PCR	Polymerase chain reaction
PI3K	Phosphoinositol-3-kinase
PTEN	phosphatase and tensin homolog
TGF-β	Transforming growth factor beta
TNM	Tumor-Node-Metastasis
